# Associations between *Interleukin-31* Gene Polymorphisms and Dilated Cardiomyopathy in a Chinese Population

**DOI:** 10.1155/2017/4191365

**Published:** 2017-05-10

**Authors:** Huizi Song, Ying Peng, Bin Zhou, Nan Chen, Xiaochuan Xie, Qingyu Dou, Yue Zhong, Li Rao

**Affiliations:** ^1^Department of Cardiology, West China Hospital of Sichuan University, Chengdu 610041, China; ^2^West China School of Medicine/West China Hospital of Sichuan University, Chengdu 610041, China; ^3^Laboratory of Molecular Translational Medicine, Key Laboratory of Obstetric & Gynecology and Pediatric Diseases and Birth Defects of Ministry of Education, West China Institute of Women's and Children's Health/West China Second University Hospital, Sichuan University, Chengdu, Sichuan, China

## Abstract

To explore the role of *Interkeulin-31 (IL-31)* in dilated cardiomyopathy (DCM), in our study, two SNPs of *IL-31*, rs4758680 (C/A) and rs7977932 (C/G), were analyzed in 331 DCM patients and 493 controls in a Chinese Han population. The frequencies of C allele and CC genotype of rs4758680 were significantly increased in DCM patients (*P* = 0.005, *P* = 0.001, resp.). Compared to CC genotype of rs4758680, the A carriers (CA/AA genotypes) were the protect factors in DCM susceptibility while the frequencies of CA/AA genotypes were decreased in the dominant model for DCM group (*P* < 0.001, OR = 0.56, 95%CI = 0.39–0.79). Moreover, IL-31 mRNA expression level of white blood cells was increased in DCM patients (0.072 (0.044–0.144) versus 0.036 (0.020–0.052), *P* < 0.001). In survival analysis of 159 DCM patients, Kaplan-Meier curve revealed the correlation between CC homozygote of rs4758680 and worse prognosis for DCM group (*P* = 0.005). Compared to CC genotype, the CA/AA genotypes were the independent factors in both univariate (HR = 0.530, 95%CI = 0.337–0.834, *P* = 0.006) and multivariate analyses after age, gender, left ventricular end-diastolic diameter, and left ventricular ejection fraction adjusted (HR = 0.548, 95%CI = 0.345–0.869, *P* = 0.011). Thus, we concluded that *IL-31* gene polymorphisms were tightly associated with DCM susceptibility and contributed to worse prognosis in DCM patients.

## 1. Introduction

Dilated cardiomyopathy (DCM) as a primary myocardial disease is marked by dilation of the left ventricle as well as systolic dysfunction that is with progressive functional and structural changes [1, 2]. It affects −1/2500 adults and more common in men than in women [3, 4]. DCM is one of the pivotal causes of sudden cardiac death and congestive heart failure concurrent with the main indication for heart transplantation [5, 6]. Over the last years, substantial studies have focused on the etiology and development of DCM, whereas the exact cause of DCM was still not understood. Increasing evidence supports several cytokines implicated in the inflammatory, and immune responses are participating in the pathological process of DCM even congestive heart failure [7, 8]. It has been delineated that the gene polymorphisms of proinflammatory cytokines such as interleukin- (IL-) 6 and tumor necrosis factor-*α* (TNF-*α*) are associated with the susceptibility and prognosis of DCM or heart failure [7–9].

Interleukin-31 (IL-31) is a novelly detected proinflammatory cytokine belonging to gp130/IL-6 cytokine family which includes IL-6, IL-11, IL-27, oncostatin M, cardiotrophin-1, cardiotrophin-like cytokine, and leukemia inhibitory factor [10, 11]. It is produced principally by the activated CD4^+^ T cells, especially when cells are skewed toward a Th2 phenotype [10, 12]. Distinguished with gp130 family, it acts through a heterodimeric receptor consisting of IL-31RA which is gp130-like receptor and oncostatin receptor (OSMR) [10]. As previously reported, IL-31 significantly stimulated the secretion of proinflammatory cytokines, such as IL-6 from monocytes and macrophages [13] and human colonic subepithelial myofibroblasts [14]. IL-6 could induce a hypertrophic response in myocytes [15], and TNF-*α* could trigger the left ventricular dilation [16]. In addition, the available data exhibited that IL-31 contributed to atopic dermatitis [17, 18], nonatopic eczema [19], systemic lupus erythematosus (SLE) [20], asthma [21], inflammatory bowel disease (IBD) [14], familial primary cutaneous amyloidosis [22], Kawasaki disease [23], hepatitis B virus liver failure [24], and allergic rhinitis [25]. These observations imply that IL-31 may contribute to the pathogenesis of DCM via cytokine modulation of immune response.

However, thus far, no study on the correlation between *IL-31* and DCM was reported. Therefore, we conducted the pilot study to clarify the role of *IL-31* in DCM patients in a Chinese population.

## 2. Materials and Methods

### 2.1. Study Subjects

The case group contained 331 subjects (male/female: 214/117, mean age: 50.16 ± 14.01 years) diagnosed as DCM recruited from the West China Hospital from June 2002 to October 2015. Since the median of the left ventricular ejection fraction (LVEF) among DCM patients was 30%, DCM patients were divided into two groups (LVEF < 30% versus LVEF ≥ 30%) in SNP-stratified analysis. The diagnosis of DCM was made in consistent with the criteria established by the World Health Organization/International Society and Federation of Cardiology Task Force on the Classification of Cardiomyopathies in 1995 (before 2006) and the scientific statement on the definitions and classification of cardiomyopathies proposed by the American Heart Association in 2006 (after 2006) [2, 26]. Meanwhile, for comparison, we recruited the control group from a routine health survey, and finally, 493 healthy unrelated individuals (male/female: 312/181, mean age: 49.15 ± 8.82 years) were consecutively enrolled. The patients with hypertension, coronary heart disease, cardiac valve disease, tachyarrhythmia, acute viral myocarditis, heavy alcohol intake, skeletal myopathies, systemic diseases of putative autoimmune origin, diabetes, and obesity or insulin resistance were excluded from the study. Written informed consents were obtained from all included subjects, sequentially, and 10 mL of peripheral venous blood was drawn from each of the DCM patient and control subjects. The present study was approved by the hospital ethics committee.

### 2.2. Extraction of DNA and Genotyping

Genomic DNA was extracted from 200 *μ*l EDTA-anticoagulated peripheral blood sample with a DNA isolation kit (BioTeke, Peking, China) as the manufacturer's direction. DNA was stably stored at −20°C until assayed. Genotyping of the *IL-31* gene polymorphism was conducted by polymerase chain reaction-restriction fragment length polymorphism (PCR-RFLP). We designed the PCR primers with software Primer 3 (http://bioinfo.ut.ee/primer3‐0.4.0/primer3/) [27] as shown in Table 1. The 10 *μ*l PCR reaction system was consisted of 1.0 *μ*l DNA and 5 *μ*l 2× Power Taq PCR Master Mix (BioTeke, Peking, China), forward and reverse primer 0.1 *μ*l, respectively, and reserved volume was made up to 10 *μ*l by sterilized water. The PCR condition was designed as 95°C for 4 min firstly, then 33 cycles at 95°C for 30 s, 60°C for 30 s, and 72°C for 30 s, and finally, 72°C for 10 min. Furthermore, the PCR products were digested in 37°C stable incubation by distinguished restriction enzyme MboII (New England Biolabs, Peking, China) for 30 minutes of rs4758680 and ScrFI (New England Biolabs, Peking, China) for 2 hours of rs7977932 as shown in Table 1, separately. Ultimately, the results were visually analyzed by 6% polyacrylamide gels in silver staining. To verify the genotyping results, DNA sequencing was performed in about 20% PCR-amplified DNA samples randomly.

### 2.3. mRNA Isolation, Reverse Transcription, and Quantitative Real-Time PCR (qPCR)

Quantitative real-time PCR of IL-31 was conducted in 41 DCM patients and 49 controls. Total RNA was isolated from white blood cells (WBCs) with TRIzol reagent (Invitrogen, Karlsruhe, Germany) and then was reverse-transcribed into cDNA, using the Bioneer kit (R&D Center, Korea) following the manufacturer's protocols. Actin was chosen as a reference parameter to normalize the data. The sense and anti-sense primers for *IL-31* and *Actin* were 5′-CTCACTCAGGCCCCTCGAC-3′ and 5′-GTCGTAGTAAACGGACGGGC-3′, 5′-TGACGTGGACATCCGCAAAG-3′ and 5′-CTGGAAGGTGGACAGCGAGG-3′, respectively.

The qPCR settings in triplicate carried on a MasterCycler realplex^4^ (Eppendorf, Wesseling-Berzforf, Germany) using SYBR Green were as follows: an initial activation step of 10 min at 95°C, subsequently by two-step cycling for 25 times: denaturation of 15 s at 94°C, and annealing and extension of 20 s at 60°C. Melting curve was added to check the amplification specificity. 2^−△ct^ method was used to calculate the mRNA expression levels [28].

### 2.4. Patients' Clinical Characteristics and Follow-Up

One hundred and fifty-nine DCM patients who have reserved contact information were brought into follow-up plan every three months until September 13, 2016. Basic clinical materials of enrolled DCM patients were obtained from the medical records (age, gender, etc.) and echocardiographic measurement using a S5-1 broadband phased-array transducer (1–5 MHz). According to the recommendations of the American Society of Echocardiography, we conducted a comprehensive 2D and Doppler echocardiography. The echocardiographic indicators such as the left ventricular end-diastolic diameter (LVEDD) were calculated with M-mode echocardiography with the left parasternal window while the left ventricular ejection fraction (LVEF) was accessed by apical two- and four-chamber views with the modified Simpson rule. The follow-up end point was patient's death because of heart failure or sudden cardiac events. A blind manner about patient's genetic status was applied during clinical follow-up.

### 2.5. Statistical Analysis

Quantitative variables were presented as median and interquartile range (IQR: Q25%–Q75%) and categorical variables as number of observations. Normality was tested using Shapiro-Wilk's test for normality. Differences between two independent samples for continuous data were analyzed using the Mann–Whitney *U* test since the distributions were different from normal. For categorical variables, statistical analysis was based on Pearson's chi-square test.

The allelic and genotype frequencies were obtained by number counting. The differences of genotypes between the DCM and control groups including codominant, dominant, recessive, and overdominant genetic models were analyzed by using SNPStats online program; meanwhile, odds ratio and 95% confidence intervals were obtained accordingly [29].

Allelic association and Hardy-Weinberg equilibrium were assessed with chi-square test. IL-31 WBC mRNA expression level was compared using the Mann–Whitney test (for two independent groups) and Kruskal-Wallis H test with multiple comparisons post hoc tests according to the results from Shapiro-Wilk's test for normality.

Kaplan-Meier curve and Cox proportional hazard models were applied to evaluate the role of IL-31 SNPs on prognosis in DCM patients. *P* value less than 0.05 was regarded as statistically significant.

## 3. Results

### 3.1. Baseline Characteristics of DCM Patients and the Controls

As shown in Table 2, between the DCM patients and the controls, gender did not exhibit statistically significant differences (*P* = 0.689). Compared to controls, DCM patients were older (*P* = 0.022) and had lower systolic blood pressure (SBP), diastolic blood pressure (DBP), left ventricular ejection fraction (LVEF), higher left ventricular end-diastolic diameter (LVEDD), and brain natriuretic peptide (BNP) (*P* < 0.001, resp.) as well as more severe NYHA functional class (*P* < 0.001). All DCM patients accepted medication treatment according to the clinical guidelines for DCM and heart failure.

### 3.2. Associations between IL-31 SNPs and Susceptibility for DCM and DCM Patients' Characteristics

The gene polymorphisms of *IL-31* rs4758680 and rs7977932 were identified to compare the allelic and genotype frequencies of 331 DCM patients to 493 controls through direct counting. About 20% PCR-amplified DNA samples were randomly evaluated by DNA sequencing, and the PCR-RFLP results were manifested as 100% accurate. The distribution of both rs4758680 and rs7977932 alleles in control groups was line with the postulation of Hardy-Weinberg equilibrium (*χ*^2^ = 1.876, *P* = 0.17 for rs4758680; *χ*^2^ = 0.004, *P* = 0.95 for rs7977932).

As shown in Table 3, the strikingly statistical difference was discovered at rs4758680. The allele frequency of C of SNP rs4758680 in DCM patients was significantly elevated compared with that in controls (89.6% versus 84.8%); in contrast, the allele A frequency was declined (10.4% versus 15.2%, *P* = 0.005, OR = 0.65, 95%CI = 0.48–0.88) in case group. In codominant model, the frequencies of the CC, CA, and AA genotypes of rs4758680 were 83.1%, 13.0%, and 3.9% in cases and were 73.2%, 23.1%, and 3.6% in controls, respectively. The differences among genotype frequencies were statistically significant (*P* = 0.001). In dominant model, compared with CC genotype, a notably decreased DCM risk was related with CA/AA genotypes (*P* < 0.001, OR = 0.56, 95%CI = 0.39–0.79). Subjects with heterozygous genotype (CA genotype) of rs4758680 had distinctly decreased risks for DCM compared with the CC/AA genotypes in overdominant model (*P* < 0.001, OR = 0.50, 95%CI = 0.34–0.73). As shown in Table 3, there was no significant difference described between DCM patients and controls in rs7977932 gene polymorphism analysis. To provide insights into the effects of *IL-31* SNPs on DCM, we exhibited the stratified analyses among 331 DCM patients. After adjusted by age and gender, the association between rs4758680 of *IL-31* and LVEF was shown in Table 4 which revealed that the heterozygote CA was the protect factor for DCM patients whose LVEF was <30% compared with those LVEF was ≥30% (*P* = 0.042). There were no statistically significant differences between the two SNPs of *IL-31* (rs4758680 and rs7977932) and LVEDD.

### 3.3. IL-31 WBC mRNA Expression Level

**Figure 1:**
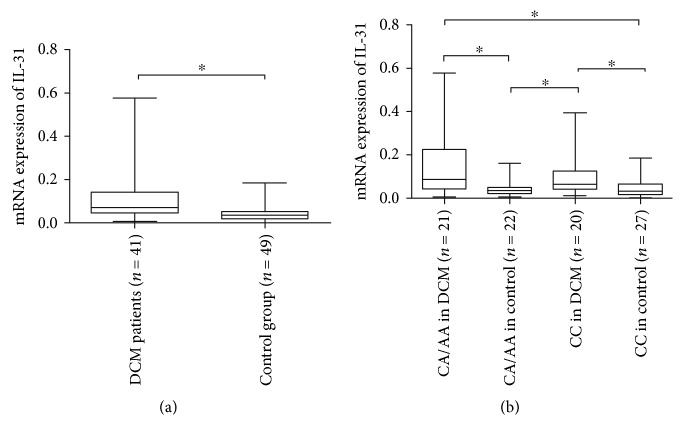
Comparsion of WBC IL-31 mRNA expression level between the DCM patients and the control group (a); comparsions of WBC IL-31 mRNA expression levels among rs4758680 different genotypes (b). The results were presented on box plots (median, IQR, range), ^∗^*P* < 0.001.

The median and IQR of 2^−△ct^ result among 41 DCM patients were 0.072 (0.044–0.144), while it was 0.036 (0.020–0.052) among 49 controls, and the difference for IL-31 WBC mRNA expression between DCM group (*n* = 41) and control group (*n* = 49) was statistically significant (*P* < 0.001) as shown in Figure 1(a). To probe into the functional influence of the IL-31 (rs4758680) genotype polymorphism (CC, CA, and AA genotypes) on the IL-31 WBC mRNA expression, the quantitative IL-31 mRNA results among different genotypes were analyzed. The IL-31 mRNA results for CA/AA genotypes in DCM (*n* = 21) and controls (*n* = 22) were 0.087 (0.044–0.223) and 0.038 (0.021–0.050), and those for CC genotype in DCM (*n* = 20) and controls (*n* = 27) were 0.065 (0.044–0.124) and 0.033 (0.018–0.065). As Figure 1(b) delineated, the *P* value was less than 0.001 for Kruskal-Wallis H test and the results of multiple comparisons post hoc tests revealed that there were statistically significant differences for IL-31 mRNA level between CA/AA genotypes in DCM and CA/AA genotypes in controls, CC genotype in DCM and CA/AA genotypes in controls, CA/AA genotypes in DCM and CC genotype in controls, and CC genotype in DCM and CC genotype in controls (*P* < 0.001, resp.). There were no statistically significant differences between CA/AA genotypes and CC genotype in DCM, as well as in controls (*P* = 0.191, 0.389, resp.).

### 3.4. Survival Analysis of IL-31 Genotypes in DCM Patients

The prognosis of DCM associated with two SNPs of *IL-31* gene was carried out by survival analysis. 159 DCM patients (mean age, 51.03 ± 13.43 years; male/female, 107/52) were tracked for a mean period of three months. 20 DCM patients were lost during the follow-up. During the follow-up, all included patients accepted consecutive medication treatment and none underwent heart transplantation.

When ending up the follow-up, 104 (65.4%) DCM patients died ascribable to cardiac events. The baseline characteristic differences between 104 dead DCM patients and 35 survival DCM patients were described in Supplementary Table available online at https://doi.org/10.1155/2017/4191365, and there were no statistically significant differences between the two analyzed groups for gender, SBP, DBP, and LVEDD (*P* = 0.251, 0.789, 0.431, and 0.817, resp.). By contrast, dead DCM patients were younger (*P* = 0.036) and had worse NYHA functional class (*P* < 0.001), lower LVEF (*P* = 0.025), and higher BNP (*P* = 0.020).

**Figure 2:**
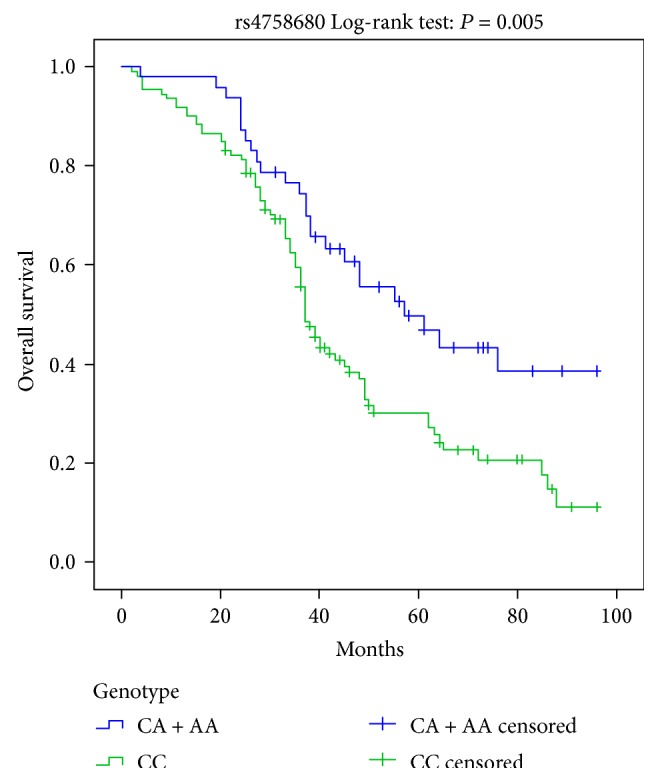
Kaplan-Meier survival curves for the dominant model of IL-31 rs4758680 polymorphism.

**Figure 3:**
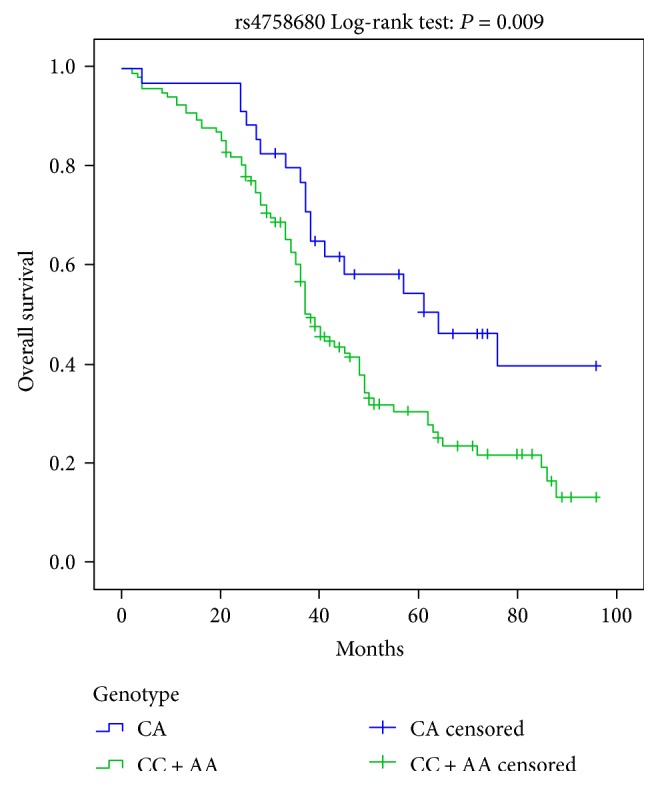
Kaplan-Meier survival curves for the overdominant model of IL-31 rs4758680 polymorphism.

Kaplan-Meier curves indicated that CC homozygote and CC/AA genotypes of *IL-31* rs4758680 were implicated in worse prognosis for DCM patients, respectively (Log-rank: *P* = 0.005, Figure 2; Log-rank: *P* = 0.009, Figure 3). Cox univariate survival analysis revealed A carriers (CA/AA genotypes) were correlated with better prognosis compared to CC genotype in genetic dominant model for DCM patients (HR = 0.530, 95%CI = 0.337–0.834, *P* = 0.006, Table 5). Similarly in overdominant model of rs4758680, compared to CC and AA homozygotes, heterozygote CA accounted for better prognosis for DCM patients (HR = 0.516, 95%CI = 0.310–0.861, *P* = 0.011, Table 5). After adjusting for age, gender, LVEDD, and LVEF, the associations between A carriers (CA/AA genotypes), CA heterozygote of rs4758680, and prognosis of DCM were still statistical significant for both dominant and overdominant models in multivariate Cox proportional hazard model analysis (HR = 0.548, 95%CI = 0.345–0.869, *P* = 0.011 and HR = 0.503, 95%CI = 0.297–0.852, *P* = 0.011, Table 5, resp.). There were no statistical differences between *IL-31* rs4758680 recessive model as well as rs7977932 gene polymorphism and overall survival time in univariate and multivariate Cox proportional hazard models as shown in Table 5.

## 4. Discussion

As previously reported, although the etiology and pathogenesis of DCM were complicated, chronic inflammation might contribute to cardiac remodeling and the development of DCM [30], and abnormal immune responses were proposed to be prominent factors in the DCM process especially after myocarditis [31]. Inflammatory cytokines like IL-6 in conjunction with TNF-*α* participated in myocyte apoptosis and myofibrosis which were involved in DCM [15, 32].

Beyond that, the established studies validated that IL-31 as a novel inflammatory cytokine was a potent inducer of proinflammatory mediators such as IL-6 in various cell types, including epithelial cells, colonic subepithelial myofibroblasts, PBMCs, macrophages, and eosinophils [33]. Matrix metalloproteinases (MMPs) also can be induced by IL-31 in colonic subepithelial myofibroblasts of IBD [14] and their higher serum level was involved in continuous extracellular matrix remodeling and increased collagen turnover of DCM with mildly dilated left ventricle [34, 35]. Shen et al. revealed that inhibition of MMPs, especially MMP-2, could reduce apoptosis from TNF-*α* in cultured cardiac myocytes [36]. In the past few years, IL-31 receptor was principally found in the skin, brain, lung, skeletal muscle, ovary, testis, prostate, spleen, thymus, bone marrow, and more [10]. In coincident with IL-31 receptor distribution, IL-31 was already identified to be associated with immune-dysfunction diseases such as atopic dermatitis, SLE, and asthma.

The established studies suggested that soluble IL-31RA might expand the range of responsive cells and tissues because of the transsignaling for IL-6 [37]; meanwhile, myocarditis had overlapping loci with diabetes and SLE, suggesting that these autoimmune diseases shared genetic traits [1]. Similarly, Doria et al. identified that DCM was one of the most serious complications involved in SLE [38] and D. Y. Chen et al. have verified that the level of Th17-related cytokines (containing IL-6, TNF-*α*) was elevated in SLE-related DCM [39]. These inferences imply a possible role of IL-31 in immune response and in DCM pathogenesis process.

The present study was the first one to investigate the correlation between IL-31 and DCM in a Han Chinese population. The human IL-31 gene is located on chromosome 12q24.31 and encodes a protein with 164 amino acids. Both rs4758680 (C/A) and rs7977932 (C/G) are in chromosome 12 intron region of *Homo sapiens* which have been implicated in SLE and AD [19, 20, 40].

Our results showed that genotype frequencies in the codominant, dominant, and overdominant models of rs4758680 were associated with DCM susceptibility. The C allele frequency of rs4758680 (C/A) in DCM was elevated, whereas the A allele was declined. We manifested that the C allele was the main predisposing factor and A carriers (CA/AA genotypes) were the protect factors for DCM especially in genetic dominant model. The CC genotype frequency of rs4758680 of *IL-31* was also relevance with DCM worse prognosis in Kaplan-Meier curve and Cox proportional hazard models. Moreover, in accordance with SNP results of rs4758680, the IL-31 WBC mRNA expression level was overtly elevated in DCM group, and the WBC mRNA expression levels of CC and CA/AA genotypes in DCM patients were higher than those in control group. As previously reported, IL-31 acted through the receptor complex of IL-31RA which is gp130-like receptor and OSMR-*β*. gp130 has been shown to mediate the cardiotrophin-1 (CT-1) in the heart that resulted in LV hypertrophy [41]. OSMR-*β* has been proved as increasing expression trend in DCM patients [42] and signaling in myocardium that result in loss of sarcomere elements and cardiac fibroblast in mouse cardiac fibroblasts [43]. Kunsleben et al. delineated that the calcium influx was induced by IL-31 in eosinophils mainly through OSMR mediating, which prompt IL-31 may affect the myocardial contraction [44]. Hence, we concluded that rs4758680 of *IL-31* SNPs played a pathogenic role in DCM patients by facilitating IL-31 protein production. In our study, the number of WBC mRNA samples was not large enough, especially the number of AA genotype was only three cases and two cases in DCM and controls, respectively. The precise and intricate mechanisms for protein expression were still unclear, and much more studies will be indispensable. By contrast, the genotype frequency of rs7977932 was absence in the DCM susceptibility. As previously reported, rs7977932 (C/G) genotype polymorphism of *IL-31* was implicated in SLE and AD [20, 40]. The difference of inclusive quantity or disease essence between our study in DCM and the previous one in SLE may account for the inconsistency of rs7977932 effect.

## 5. Conclusions

In conclusion, IL-31 as scratch factor was a research hot spot before we firstly revealed that rs4758680 (C/A) of *IL-31* was associated with the susceptibility of DCM in the Chinese Han people although larger sample sizes of *IL-31* SNP would be necessary to confirm our findings; moreover, CC genotype was implicated in the worse prognosis in DCM group. Even so, plasma IL-31 protein level and the underlying mechanisms were lack in our study; besides, more SNPs of *IL-31* with DCM susceptibility and prognosis in a variety of ethnic populations need to be investigated in future studies.

## Supplementary Material

Supplementary Table. The baseline characteristic differences between dead DCM patients and survival DCM patients.

## Figures and Tables

**Table 1:** Information about PCR-RFLP in DCM and control groups.

SNP ID	Primer sequence	Major/minor gene	Product (bp)	Annealing temperature (°C)	Restriction enzyme	Allele (bp)
rs4758680	F:5′-GATCACCCGGACTCAAAACGTG-3'	C/A	263	60	MboII	A (263)
	R: 5′-TTGTGCAAACCACACCTCTTCG-3'	—	—	—	—	C (210 + 53)

rs7977932	F:5′-GGTCAGTGTTGGGTTTGCAATG-3'	C/G	121	60	ScrFI	G (74 + 57)
	R:5′-TTGGTGATGGCACAGCCTCATA-3'	—	—	—	—	C (131)

**Table 2:** Baseline characteristics of the DCM patients and the controls.

Variables	DCM patients (*n* = 331)	Controls (*n* = 493)	*P* value
Age (years)	52 (43–60)	50 (42–57)	0.022
Gender (male/female)	214/117	312/181	0.689
SBP (mmHg)	116 (109–123)	121 (114–127)	<0.001
DBP (mmHg)	73 (68–77)	77 (72–81)	<0.001
NYHA	II: 62; III: 210; IV: 59	I: 395; II: 98	<0.001
LVEDD (mm)	67 (62–73)	46 (44–49)	<0.001
LVEF (%)	30 (25–37)	62 (58–66)	<0.001
BNP (pg/ml)	2787 (1600–3836)	95 (83–108)	<0.001

Data are exhibited as the median ± interquartile range(IQR: Q25%–Q75%) or number; DCM: dilated cardiomyopathy; SBP: systolic blood pressure; DBP: diastolic blood pressure; NYHA: New York Heart Association; LVEDD: left ventricular end-diastolic diameter; LVEF: left ventricular ejection fraction; BNP: brain natriuretic peptide.

**Table 3:** Distributions of *IL-31* SNPs among cases and controls and their associations with DCM susceptibility.

	Genotype	rs4758680				Genotype	rs7977932			
		Cases *n* (%)	Controls *n* (%)	OR (95%CI)	*P* value		Cases *n* (%)	Controls *n* (%)	OR (95%CI)	*P* value
Model										
Codominant	CC	275 (83.1%)	361 (73.2%)	1.00	—	CC	271 (81.9%)	401 (81.3%)	1.00	—
	CA	43 (13.0%)	114 (23.1%)	**0.50 (0.34–0.72)**	**0.001**	CG	56 (16.9%)	87 (17.6%)	1.05 (0.73–1.52)	0.930
	AA	13 (3.9%)	18 (3.6%)	0.95 (0.46–1.96)	—	GG	4 (1.2%)	5 (1.0%)	0.84 (0.22–3.17)	—

Dominant	CC	275 (83.1%)	361 (73.2%)	1.00	—	CC	271 (81.9%)	401 (81.3%)	1.00	—
	CA/AA	56 (16.9%)	132 (26.8%)	**0.56 (0.39–0.79)**	**<0.001**	CG/GG	60 (18.1%)	92 (18.7%)	1.04 (0.72–1.49)	0.850

Recessive	CC/CA	318 (96.1%)	475 (96.3%)	1.00	—	CC/CG	327 (98.8%)	488 (99.0%)	1.00	—
	AA	13 (3.9%)	18 (3.6%)	0.93 (0.45–1.92)	0.840	GG	4 (1.2%)	5 (1.0%)	0.84 (0.22–3.14)	0.790

Overdominant	CC/AA	288 (87.0%)	379 (76.9%)	1.00	—	CC/GG	275 (83.1%)	406 (82.4%)	1.00	—
	CA	43 (13.0%)	114 (23.1%)	**0.50 (0.34–0.73)**	**<0.001**	CG	56 (16.9%)	87 (17.6%)	1.05 (0.73–1.52)	0.790
	Allele					Allele				
	C	593 (89.6%)	836 (84.8%)	1.00	—	C	598 (90.3%)	889 (90.2%)	1.00	—
	A	69 (10.4%)	150 (15.2%)	**0.65 (0.48–0.88)**	**0.005**	G	64 (9.7%)	97 (9.8%)	1.02 (0.73–1.42)	0.909

OR: odds ratio; CI: confidence interval; SNP analysis adjusted for age, gender, LVEDD, and LVEF.

**Table 4:** Associations between *IL-31* SNPs and DCM patients' characteristics.

	Genotype	rs4758680				Genotype	rs7977932			
		LVEF <30% *n* (%)	LVEF ≥30% *n* (%)	OR (95%CI)	*P* value		LVEF <30% *n* (%)	LVEF ≥30% *n* (%)	OR (95%CI)	*P* value
Model										
Codominant	CC	131 (85.1%)	144 (81.4%)	1.00	—	CC	128 (83.1%)	143 (80.8%)	1.00	—
	CA	14 (9.1%)	29 (16.4%)	0.52 (0.26–1.03)	0.035	CG	24 (15.6%)	32 (18.1%)	1.20 (0.67–2.15)	0.810
	AA	9 (5.8%)	4 (2.3%)	2.56 (0.76–8.33)	—	GG	2 (1.3%)	2 (1.1%)	0.82 (0.11–5.97)	—

Dominant	CC	131 (85.1%)	144 (81.4%)	1.00	—	CC	128 (83.1%)	143 (80.8%)	1.00	—
	CA/AA	23 (14.9%)	33 (18.6%)	0.76 (0.42–1.37)	0.360	CG/GG	26 (16.9%)	34 (19.2%)	1.17 (0.66–2.07)	0.590

Recessive	CC/CA	145 (94.2%)	173 (97.7%)	1.00	—	CC/CG	152 (98.7%)	175 (98.9%)	1.00	—
	AA	9 (5.8%)	4 (2.3%)	2.78 (0.84–9.09)	0.081	GG	2 (1.3%)	2 (1.1%)	0.79 (0.11–5.75)	0.820

Overdominant	CC/AA	140 (90.9%)	148 (83.6%)	1.00	—	CC/GG	130 (84.4%)	145 (81.9%)	1.00	—
	CA	14 (9.1%)	29 (16.4%)	**0.50 (0.25–0.99)**	**0.042**	CG	24 (15.6%)	32 (18.1%)	1.20 (0.67–2.16)	0.530
	Allele					Allele				
	C	276 (90.0%)	317 (90.0%)	1.00	—	C	280 (91.0%)	318 (90.0%)	1.00	—
	A	32 (10.0%)	37 (10.0%)	1.00 (0.64–1.56)	0.99	G	28 (9.0%)	36 (10.0%)	1.12 (0.67–1.88)	0.670

LVEF: left ventricular ejection fraction; OR: odds ratio; CI: confidence interval; SNP analysis adjusted for age and gender.

**Table 5:** Associations between *IL-31* SNPs and patients' overall survival.

Characteristics	Genotype	Overall survival					
		Multivariate survival analysis^a^			Univariate survival analysis		
		HR	95%CI	*P* value	HR	95%CI	*P* value
Model							
rs4758680							
Dominant	CC	1	—	—	1	—	—
	CA/AA	**0.548**	**0.345–0.869**	**0.011**	**0.530**	**0.337–0.834**	**0.006**
Recessive	CC/CA	1	—	—	1	—	—
	AA	0.868	0.399–1.888	0.722	0.778	0.361–1.678	0.522
Overdominant	CC/AA	1	—	—	1	—	—
	CA	**0.503**	**0.297–0.852**	**0.011**	**0.516**	**0.310–0.861**	**0.011**
rs7977932							
Dominant	CC	1	—	—	1	—	—
	CG/GG	1.161	0.666–2.024	0.599	1.275	0.736–2.211	0.386
Recessive	CC/CG	1	—	—	1	—	—
	GG	3.739	0.862–16.218	0.078	3.482	0.841–14.414	0.085
Overdominant	CC/GG	1	—	—	1	—	—
	CG	1.096	0.619–1.943	0.753	1.211	0.687–2.133	0.508

^a^Multivariate survival analysis adjusted for age, gender, LVEDD, and LVEF.
