# Supplementary material for: Associations between Interleukin-31 Gene Polymorphisms and Dilated Cardiomyopathy in a Chinese Population
Source: Dis Markers. 2017 May 10;2017:4191365. doi: 10.1155/2017/4191365 (PMC5442432; doi:10.1155/2017/4191365)
Supplement: Supplementary file 1 — Supplementary Table. The baseline characteristic differences between dead DCM patients and survival DCM patients. [file 4191365.f1.docx]

| Variables | Dead DCM (n=104) | Survival DCM (n=35) | *p* value |
| --- | --- | --- | --- |
| Age (years) | 51(40-57) | 57(47-63) | 0.036 |
| Gender (male/female) | 76/28 | 22/13 | 0.251 |
| SBP (mmHg) | \| 114(107-121) \| \| --- \| | 116(109-122) | 0.789 |
| DBP (mmHg) | \| 72(68-77) \| \| --- \| | \| 73(69-78) \| \| --- \| | 0.431 |
| NYHA | II: 4; III: 57; IV: 43 | II: 21; III: 12; IV: 2 | <0.001 |
| LVEDD (mm) | 68(63-73) | 67(62-77) | 0.817 |
| LVEF (%) | 29(23-36) | 33(28-38) | 0.025 |
| BNP (pg/ml) | 2792(1794-3694) | 2222(862-3179) | 0.020 |

**Supplementary Table The baseline characteristic differences between dead DCM patients and survival DCM patients.**

Data are exhibited as the median ± interquartile range(IQR: Q25%-Q75%) or number; Abbreviations: DCM, dilated cardiomyopathy; SBP, systolic blood pressure; DBP, diastolic blood pressure; NYHA, New York Heart Association; LVEDD, left ventricular end-diastolic diameter; LVEF, left ventricular ejection fraction; BNP, brain natriuretic peptide
